# Burden of thyroid cancer in China and worldwide from 1990 to 2021: observation, comparison, and forecast from the Global Burden of Disease Study 2021

**DOI:** 10.3389/fendo.2024.1500926

**Published:** 2024-12-06

**Authors:** Ziang Meng, Ti Pan, Jingjing Yu, Chao Shi, Xuxu Liu, Dongbo Xue, Jing Wang, Biao Ma

**Affiliations:** ^1^ Department of General Surgery, The First Affiliated Hospital of Harbin Medical University, Harbin, China; ^2^ Key Laboratory of Hepatosplenic Surgery, Ministry of Education, The First Affiliated Hospital of Harbin Medical University, Harbin, China; ^3^ NHC Key Laboratory of Etiology and Epidemiology (National Health Commission of the People’s Republic of China), Harbin Medical University, Harbin, China

**Keywords:** thyroid cancer, epidemiology, age-period-cohort analysis, ARIMA model, disease burden, joinpoint regression

## Abstract

**Background:**

Thyroid cancer (TC) is a prevalent malignant tumor of the endocrine system in China. Current research primarily focuses on clinical diagnosis and treatment as well as underlying mechanisms, lacking epidemiological studies on the burden of the disease in China and worldwide.

**Methods:**

The Global Burden of Disease Study 2021 was utilized to assess the incidence, prevalence, death, and disability-adjusted life years of TC in China and worldwide from 1990 to 2021 using the Joinpoint and R software.

**Results:**

From 1990 to 2021, the incidence and prevalence rates of TC in China have been consistently rising, and their growth rates are far higher than the global average. In China, TC usually occurs in patients aged 50-59, and the crude death rate generally increases with age. The burden of death among females has gradually declined, while that among males has continued to increase and surpassed females at the beginning of the 21st century. The burden of TC is heavy among middle-aged and elderly populations and the younger populations is also rapidly rising. The increased number of TC is mainly attributed to epidemiological changes, while the increase of deaths in China is primarily due to aging and population. Additionally, we predict that the age-standardized incidence rate of TC in China will continue to grow slowly over the next decade, while the age-standardized death rate will gradually decline among females and stabilize among males.

**Conclusion:**

It is imperative to avoid over-screening and over-treatments for TC. Meanwhile, we should also avoid missing aggressive types of TC that may have an impact on overall survival. Additionally, understanding the mechanisms of metastasis and improving clinical treatments should be prioritized for further investigation. TC remains a significant public health challenge in China, necessitating a careful balance of the cost-benefit ratio.

## Introduction

Thyroid cancer (TC) represents the most prevalent malignant tumor of the endocrine system (1), ranking seventh in the global cancer incidence rate in 2024. Projections indicate that it may surpass breast cancer to become the fourth most prevalent cancer worldwide in the future (2). TC can be classified into papillary thyroid cancer (PTC), follicular thyroid cancer (FTC), medullary thyroid cancer (MTC), and anaplastic thyroid cancer (ATC). PTC and FTC are also referred to as differentiated thyroid cancer (DTC). PTC is the most common subtype of TC with the best prognosis, while ATC is the subtype with the worst prognosis. Over the past four decades, the incidence of TC has increased by 313% (3), with a notable rise in the prevalence of various pathological types of TC (4). In the United States, approximately 1.2% of the population will be diagnosed with TC at some point during their lifetime (5), It was estimated that 43,720 new cases of TC was diagnosed in 2023. In recent years, numerous studies have demonstrated that the incidence of TC persists in a rising trend in numerous countries and regions, including North America (6, 7), South America (8), Asia (9, 10), and Europe (11). Nearly half of the TC burden occurs in South and East Asia (12), with females accounting for the majority of the burden and an increasing trend over the years, despite the fact that age-standardized incidence rate (ASIR) of TC in males has increased faster than that in females. However, the underlying causes of this phenomenon remain poorly understood. Concurrently, there are discrepancies in the prevalence and impact of TC across diverse populations. The proportion of TC death contributable to high BMI was highest in developed countries and middle-aged people (13). TC is more prevalent in women than in men, with a ratio of approximately three to one in most populations (14). This gender disparity may be attributed to various factors, including sex hormones, genetics, and the immune system (15).

Many studies have indicated that approximately half of the global incidence of TC can be attributed to East Asia. The countries with the highest rates of TC are China, the United States, and India (16). This illustrates the significant influence of China on the global prevalence of TC. “Basic”, “clinical”, and “surgical” represent the three major research hotspots in TC, particularly in basic research areas such as genetics and genomics, molecular biology and signaling, and preclinical and translational science (17). However, there is a scarcity of epidemiological studies on TC, especially regarding the geographical distribution and trend changes of the disease burden. The reports on the burden of TC in Global Burden of Disease (GBD) studies primarily focus on the overall situation at the global, national, or regional level, revealing the global distribution, trends, attributable risks of TC burden, and its relationship with socio-demographic index. These studies also provide future predictions of the TC burden (18, 19). However, they do not delve into the heterogeneity between different countries and regions, particularly neglecting the specific situations of particular countries and their relationship to the global level. Additionally, there is a lack of GBD studies on the prevalence of TC.

In this study, we utilized the GBD 2021 database to analyze incidence, prevalence, death, and disability-adjusted life years (DALYs) rate or number in China and worldwide. The aim is to observe, compare, and predict the disease burden of TC in China and worldwide. The findings of our study are intended to furnish valuable insight for the formulation of pertinent national medical and health policies, the allocation of medical resources, and the planning of health systems, to reduce the burden of TC in China and worldwide, which holds significant implications for other developing countries as a reference.

## Methods

### Data acquisition and download

The disease burden indicators for TC in China and worldwide from 1990 to 2021 are derived from the GBD 2021 public database (https://ghdx.healthdata.org/gbd-results). Additionally, demographic data for China and the worldwide from 1990 to 2021 were obtained. The database study employed the most recent epidemiological data and enhanced standardized methods to report on disease-related health damage across 369 diseases in 204 countries and territories. Furthermore, the primary estimation methods employed are the Bayesian meta-regression tool DisMod-MR, the Integrated Model for the Cause of Death (CODEm), and the Spatiotemporal Gaussian Process Regression (STGPR). “China and global” was selected as the location; “1990 to 2021” as the years; “Number and Rate” as metrics; “Incidence, Prevalence, Death, and DALYs” as measures; “Male, Female, Both” as genders; “all ages, age-standardized, 0-4, 5-9, 10–14…90-94, 95+” as age and “thyroid cancer” as the cause. Additionally, The raw data used for analysis in this study can be found in the **Supplementary Material**. The ethics committee of the First Affiliated Hospital of Harbin Medical University determined that approval was not required for the study, as it utilized publicly accessible data.

### Joinpoint regression analysis

The Joinpoint regression model is a set of linear statistical models that employs the least squares method to estimate the law of change, thereby circumventing the non-objectivity inherent in typical trend analysis based on linear trends. The turning point of the moving trend is identified by calculating the sum of the squares of the residuals between the estimated and actual values. In this study, the Joinpoint regression model was employed for the purpose of assessing trends in the disease burden attributed to TC in China and worldwide. The model was created using Joinpoint software (Version 5.2.0), which was developed by the National Cancer Institute of the United States. This paper presents an analysis of trends in incidence, prevalence, deaths, and DALYs from 1990 to 2021. In order to optimize the model, the grid search method (GSM) and Monte Carlo permutation test were employed. Furthermore, the Bonferroni correction was utilized in order to maintain the overall asymptotic significance level. The results obtained include the number of inflection points, their locations, and the corresponding P-values. The annual percentage change (APC) over a specific interval is calculated by two formulas:


Ln (y) = α + β x+ ϵ

y = age-standardized rate, x = year, β is the estimated value of the slope, and ϵ was the error term.


APC=100[exp(β) – 1]

APC>0 indicated an increasing trend, APC<0 indicated a decreasing trend, APC=0 indicated no change. Average annual percentage change (AAPC) was calculated to estimate overall TC burden trend from 1990 to 2021. Finally, an APC value equal to the AAPC represents a monotonic increasing or decreasing trend. In addition to the standard non-parametric description method, the model employs log-linear regression to calculate AAPC and its 95% confidence interval (CI). Furthermore, it compares AAPC with 0 to ascertain whether the fluctuation trend of different parts is statistically significant. A statistically significant P-value was less than 0.05.

### Statistical analysis

#### R programming language

R (version 4.4.0) is a statistical computing and graph-specific language and environment that offers robust extensibility, enabling the creation of new features and the automation of analysis. All statistical analyses and visualizations of the data in this study were generated by R, and a P-value less than 0.05 was considered to be statistically significant.

In this study, the demographic variables affecting TC were included, and R packages such as “dplyr (version 1.14)”, “ggplot2 (version 3.5.1)”, “reshape2 (version 1.4.4)”, and “readxl (version 1.4.3)” were employed. The objective was to describe and visualize the distribution of TC disease burden among different age groups and genders, and to demonstrate the trend of TC burden over time in China and worldwide.

#### Age-period-cohort model

The age-period-cohort model is a frequently employed methodology in epidemiological studies. The age-period-cohort model, based on the Poisson distribution, is capable of reflecting temporal trends in incidence or death at varying ages, periods, and cohorts. The age-period-cohort model is defined by the following formula:


Y=log (M)=μ+αX1+βX2+γX3+ϵ

X1, X2, and X3 represent age, period, and birth cohort groups, respectively; M represents disease incidence or death rate; α, β, and γ denote the estimated age, period, and birth cohort effect values, respectively; μ represents the intercept; and ϵ representing random error following a normal distribution. In the present study, we employed the age-period-cohort model fitting function of the “Epi”, “source_apc”, and “function_year5” software package in R to calculate the total number of incidence or death cases and the cumulative incidence or death rate of different age groups. In order to run the age-period-cohort model in accordance with the demographic data age group, the disease burden data series for TC in China was divided into consecutive 5-year intervals from 1992 to 2021. The data from 1990 and 1991 were not included in the analysis.

#### Decomposition analysis

Decomposition analysis is a method of determining the extent to which differences in various factors contribute to differences in population values. In order to quantify the relative contribution of age structure, epidemiological changes, and population size to the burden of TC in China from 1990 to 2021, the DasGupta decomposition method was employed to visually demonstrate the role of three factors (ageing, epidemiological changes, and population) that drove changes in incidence and death of TC in China from 1990 to 2021. The term “epidemiological changes” is used to describe age-adjusted and population-adjusted incidence or death rates.

#### The autoregressive integrated moving average (ARIMA)

ARIMA model is constituted by an Autoregressive (AR) model and a Moving Average (MA) model. In instances where the time series should be a stationary random series with a mean of zero and the data series are a time-dependent random variable, the autocorrelation can be characterized by the ARIMA model, thereby enabling the prediction of future values based on past observations. In this study, the “forecast” package (version 8.23.0) was employed. The R package was used to fit the ARIMA model and plot the prediction results, with the objective of evaluating the future trend of TC incidence and death in China.

## Result

### Overview of the TC burden in China and worldwide

As illustrated in **Table 1**, the global incidence of TC has increased from 89,885 cases (95% confidence interval [CI]: 84, 681-96, 999) in 1990 to 249,538 cases (95% CI: 223, 290-274, 638) in 2021, representing a cumulative increase of 177%. A 6% increase was observed in the number of TC cases in China, rising from 12157 (95% CI: 9714-14406) in 1990 to 48105 (95% CI: 38, 695-60, 068) in 2021. This represents a cumulative increase of 295.7%, a figure that far exceeds the global increase. The global ASIR for TC increased from 2.062 (95% CI: 1.951-2.224) in 1990 to 2.914 (95% CI: 2.607-3.213) in 2021, representing a cumulative increase of 41.3%. ASIR of TC in China increased from 1.249 (95% CI: 1.009-1.473) in 1990 to 2.473 (95% CI: 1.993-3.088) in 2021, with a cumulative increase of 98%. However, it should be noted that the ASIR in China has consistently remained below the global level. Concurrently, the global incidence of TC increased by 1.1387% (95% CI: 1.0372–1.2404) from 1990 to 2021, while the incidence of TC in China increased by 2.2418% (95% CI: 2.1123–2.3714), representing a rate nearly twice that of the global level.

**Table 1:** All-age numbers and age-standardized incidence, prevalence, death, and DALYs rates and corresponding AAPC of TC in China and worldwide in 1990 and 2021.

Location	Measure	1990		2021		1990–2021 AAPC
		All-ages cases	Age-standardized rates per 100,000	All-ages cases	Age-standardized rates per 100,000	
		n (95% CI)	n (95% CI)	n (95% CI)	n (95% CI)	n (95% CI)
China	Incidence	12157 (9714–14406)	1.249 (1.009-1.473)	48105 (38695–60068)	2.473 (1.993-3.088)	2.2418 (2.1123 - 2.3714)
	Prevalence	87082 (68622–104169)	8.098 (6.41-9.66)	388411 (311967–488388)	20.012 (16.135-25.228)	2.9751 (2.833 - 3.1174)
	Deaths	3599 (3038–4182)	0.473 (0.403-0.55)	7692 (6123–9429)	0.387(0.307-0.472)	-0.6515 (-0.8237 - -0.4789)
	DALYs	110736 (92143–130509)	12.086 (10.14-14.08)	203325 (163131–251789)	10.105 (8.139-12.447)	-0.5899 (-0.787 - -0.3925)
Global	Incidence	89885 (84681–96999)	2.062(1.951-2.224)	249538 (223290–274638)	2.914 (2.607-3.213)	1.1387 (1.0372 - 1.2404)
	Prevalence	676649 (636788–727723)	14.931 (14.124-16.029)	1987148 (1776275–2198245)	23.143(20.663-25.647)	1.4184 (1.3121 - 1.5248)
	Deaths	21893 (20437–24108)	0.57 (0.53-0.628)	44799 (39925–48541)	0.53 (0.47-0.575)	-0.2297 (-0.2885 - -0.1709)
	DALYs	646741 (599118–717357)	15.206 (14.18-16.83)	1246485 (1094416–1375853)	14.571 (12.783-16.115)	-0.131 (-0.2412 - -0.0206)

In terms of prevalence, the number of TC cases worldwide increased from 676, 649 in 1990 (95% CI: 636, 788-727, 723) to 1,987,148 in 2021 (95% CI: 1, 776, 275-2, 198, 245), representing a cumulative increase of 193.7%. The number of TC in China increased from 87082 (95% CI: 68, 622-104, 169) in 1990 to 388411 (95% CI: 311, 967-488, 388) in 2021, representing a cumulative increase of 346%, which also far exceeds the global trend. The global age-standardized rate (ASPR) for TC increased from 14.931 (95% CI: 14.124-16.029) per 100,000 population in 1990 to 23.143 (95% CI: 20.663-25.647) per 100,000 population in 2021, representing a cumulative increase of 64%. In comparison, the ASPR of TC in China increased from 8.098 (95% CI: 6.41-9.66) per 100000 population in 1990 to 20.012 (95% CI: 16.135-25.228) per 100000 population in 2021, with a cumulative increase of 147.1%, which far exceeds the global increase. Concurrently, from 1990 to 2021, the global prevalence of TC increased by 1.4184% (95% CI: 1.3121–1.5248), while the prevalence of TC in China was 2.9751% (95% CI: 2.833–3.1174), which is also approximately twice the global level.

In terms of death, globally, TC was the cause of 44799 deaths (95% CI: 39, 925-48, 541) in 2021, representing a 104.6% increase from the 21, 893 deaths (95% CI: 20,437-24,108) attributed to the disease in 1990. The ASDR for TC exhibited a decline from 0.57 (95% CI: 0.53-0.628) per 100,000 population in 1990 to 0.53 (95% CI: 0.47-0.575) per 100,000 population in 2021. Similarly, the ASDR for TC in China demonstrated a reduction from 0.473 (95% CI: 0.403-0.55) per 100, 000 population in 1990 to 0.387 (95% CI: 0.307-0.472) per 100, 000 population in 2021. The AAPC reduction in TC death rate in China was -0.6515 (95% CI: -0.8237 to -0.4789), which was approximately three times the global level.

In terms of DALYs, the global number of DALYs in TC increased from 646, 741 in 1990 (95% CI: 599, 118-717, 357) to 1,246,485 in 2021 (95% CI: 1, 094, 416-1, 375, 853). The number of DALYs in TC in China increased from 110736 in 1990 (95% CI: 92, 143-130, 509) to 203, 325 in 2021 (95% CI: 163, 131-251, 789), which is not as high as that in the world. Conversely, there was a reduction in age-standardized disability-adjusted life years rate (ASDALYs) in China and worldwide, with the AAPC of DALYs in China declining by -0.5899 (95%CI: -0.787 to -0.3925), which is approximately 4.5 times the global level.

### Joinpoint regression and trend analysis of the TC burden in China and worldwide

**Figure 1:**
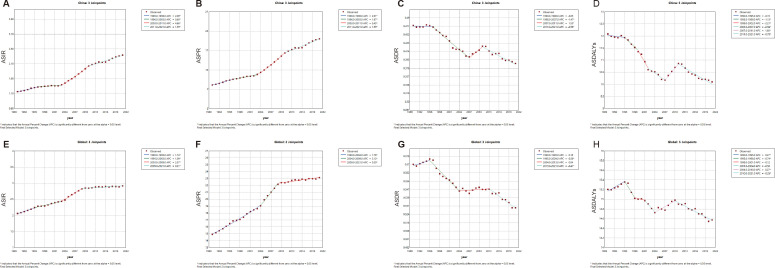
The APC of ASIR, ASPR, ASDR, and ASDALYs rates of thyroid cancer in China and Global from 1990 to 2021 (* means P-values<0.05) including **(A)** ASIR, **(B)** ASPR; **(C)** ASDR; **(D)** ASDALYs rates in China. **(E)** ASIR; **(F)** ASPR; **(G)** ASDR; **(H)** ASDALYs rates worldwide.

The combined point regression analysis of the ASIR, ASPR, ASDR, and ASDALYs for TC in China and worldwide from 1990 to 2021 is presented in **Figures 1A-H**. During this period, the ASIR and ASPR of TC in China and worldwide continued to increase. It is noteworthy that following 2003, both the ASIR and APC of the ASPR in China (ASIR: 2003-2011, APC=4.66, P<0.05; ASPR: 2003-2009, APC=5.45, P<0.05) and worldwide (ASIR: 2003-2011, APC=2.57, P<0.05; ASPR: 2004-2009, APC=3.12, P<0.05) exhibited sudden and significant upward fluctuations. In recent years, the global APC of the ASIR and ASDR has approached zero, indicating that the observed changes are not statistically significant. Nevertheless, the APCs of China’s ASIR (2009-2021 APC=1.59, P<0.05) and ASPR (2009-2021 APC=1.99, P<0.05) remain in the previous fluctuation trend. Furthermore, a downward trend was observed in the ASDR and ASDALYs of TC in China and worldwide from 1990 to 2021 (P < 0.05).

**Figure 2:**
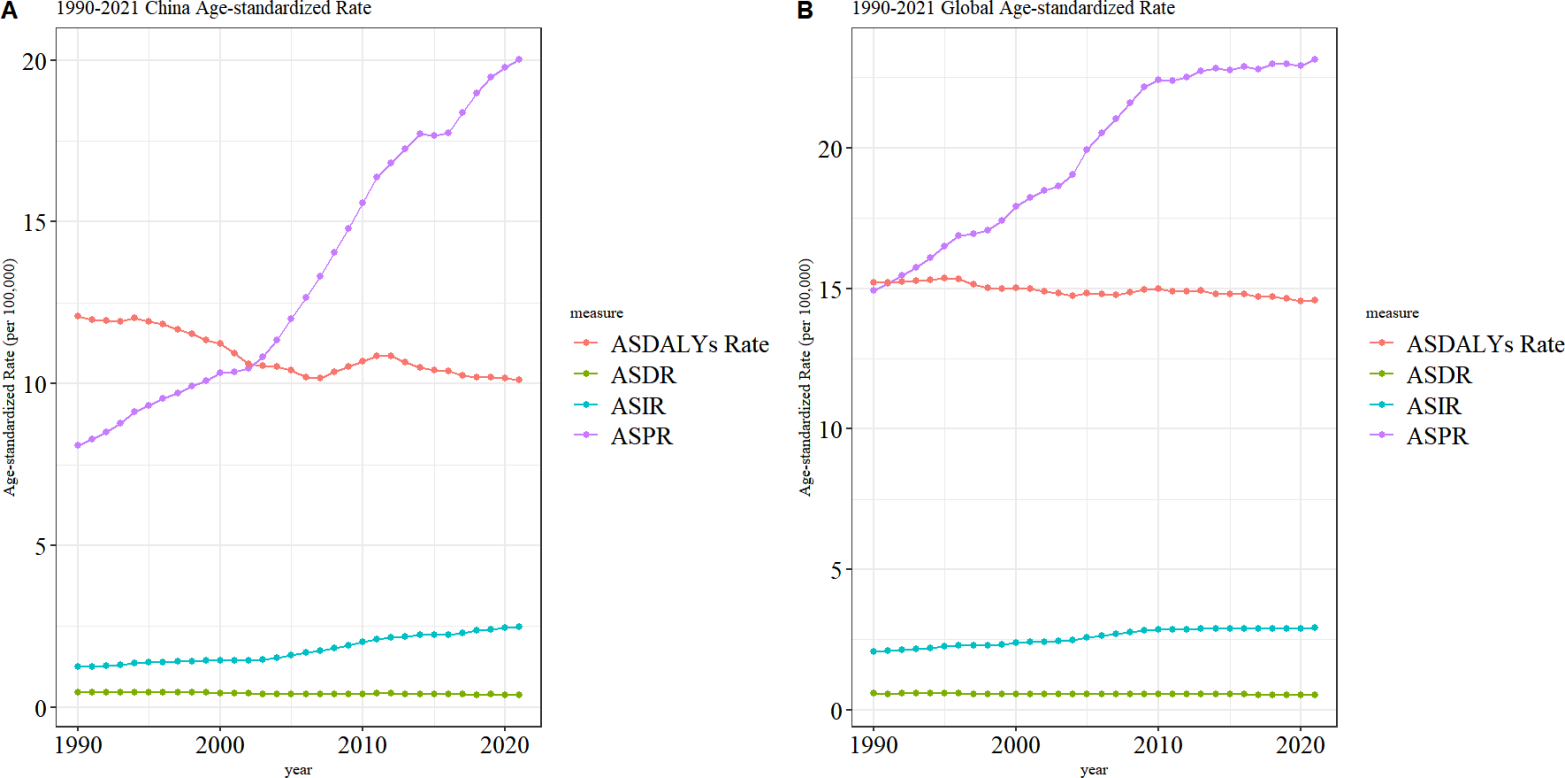
Trend of ASIR, ASPR, ASDR, and ASDALYs rates of TC in China **(A)** and worldwide **(B)** from 1990 to 2021.

As illustrated in **Figure 2**, ASIR and ASPR for TC exhibited a gradual increase in China (**Figure 2A**) and worldwide (**Figure 2B**) from 1990 to 2021. Notably, the ASPR in China demonstrated a significantly larger increase, while the ASPR stabilized gradually in recent years. The ASDR and ASDALYs rate for TC are demonstrating a gradual decline in China and worldwide. Additionally, the volatility of the ASDR in TC in China is more pronounced than that observed worldwide.

### Age differences in TC burden of China in 1990 and 2021

**Figure 3:**
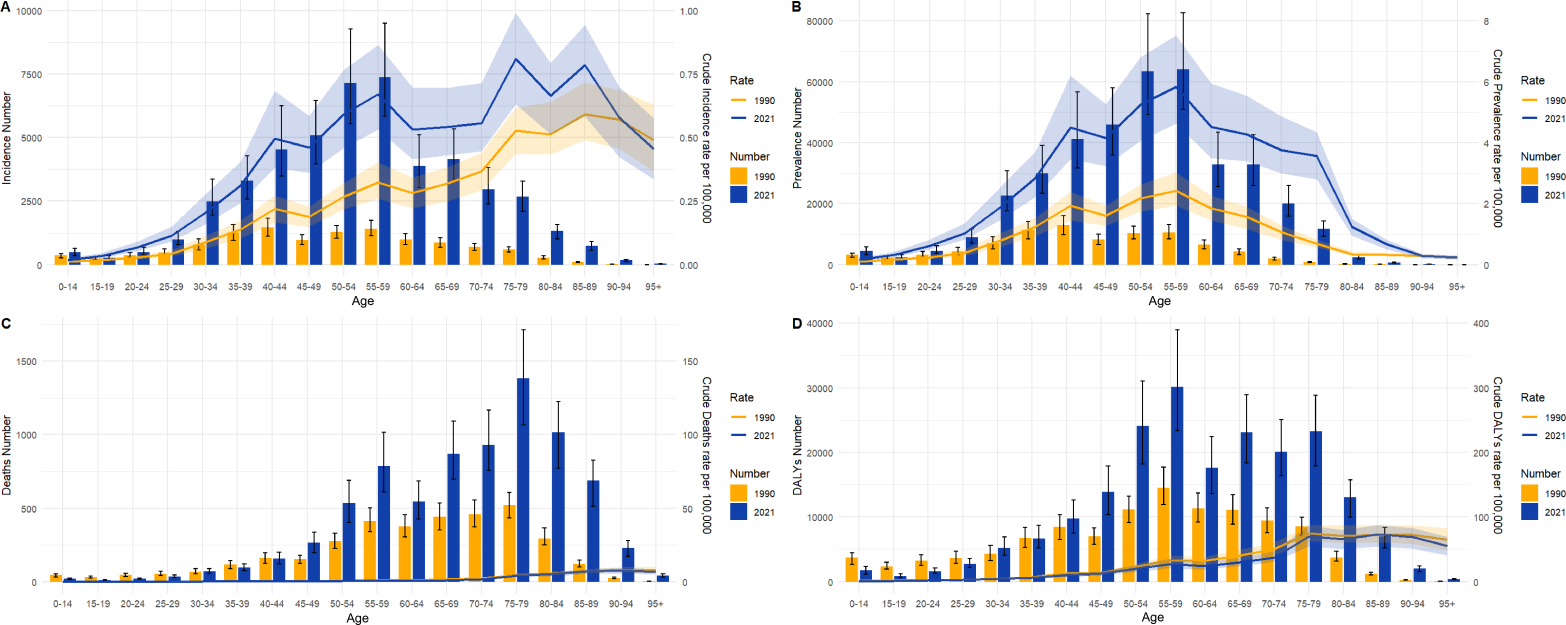
Numbers and crude rates of incidence, prevalence, death, and DALYs in China from 1990 and 2021 by age group. **(A)** Incidence number and CIR; **(B)** Prevalence number and CPR; **(C)** Death number and CDR; **(D)** DALYs and crude DALYs rate; Lines represent crude rates; Bar charts represent numbers.

**Figure 3** illustrates the incidence, prevalence, deaths, and DALYs of TC in distinct age groups of China in 1990 and 2021, accompanied by the corresponding crude rates. With regard to incidence (**Figure 3A**), TC often occurs in the 30-79 age group, with a particularly elevated incidence rate in the 50-59 age group. However, in 1990, the 40-44 age group exhibited a higher incidence than the 50-59 age group. In particular, the crude incidence rate has been observed to increase overall in all age groups. With regard to prevalence (**Figure 3B**), The crude prevalence rate and prevalence number in all age groups in 1990 and 2021 were normally distributed generally. The higher prevalence is also concentrated in the 50-59 age group. With regard to death (**Figure 3C**), the age groups with the highest number of deaths were concentrated in the 75-79 age range in both 1990 and 2021. Crude death rate generally increases with age. With regard to DALYs (**Figure 3D**), the age group with the highest number of DALYs was concentrated in both the 50-59 age range in both 1990 and 2021.

### Gender differences in TC burden among different age groups in China from 1990 to 2021

**Figure 4:**
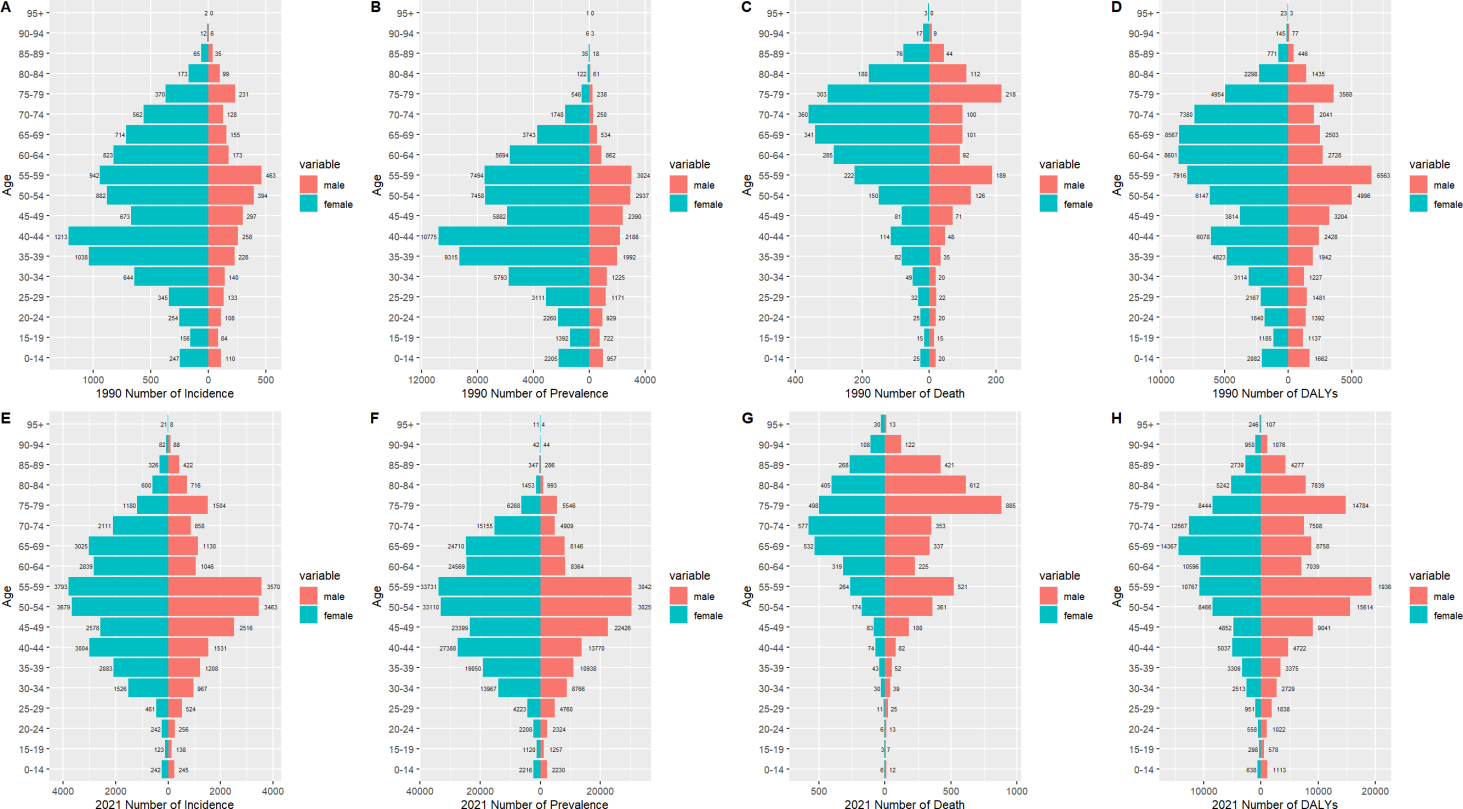
The incidence number, prevalence number, deaths number, and DALYs of TC patients in males and females of different age groups in China in 1990 **(A-D)** and 2021 **(E-H)**.

**Figure 4** illustrates the incidence, prevalence, death, and DALYs of TC in distinct age groups among men and women in China in 1990 and 2021. **Figure 4A** illustrates that the age group with the highest incidence of TC in women in 1990 was 40-44 years (n=1, 213), while that of men was 55-59 years (n=463). **Figure 4E** demonstrates that the age group with the peak incidence in 2021 was 55-59 years in both women and men (n=3,793; 3,570). Similarly, **Figure 4B** illustrates that the age group with the highest prevalence of cases in women in 1990 was 40-44 years (n=10, 775), while in men it was 55-59 years (n=3, 024). **Figure 4F** demonstrates that the age group at the peak of prevalence in 2021 was 55-59 years in both women and men (n=337, 313, 042). It is evident that in both 1990 and 2021, the number of cases in women was significantly higher than that of men in all age groups. With regard to death, **Figure 4C** illustrates that the age group with the highest number of deaths in 1990 was 70-74 years for women (n=360), while for men it was 75-79 years (n=218). **Figure 4G** illustrates that in 2021, the age group with the highest number of deaths in women was 70-74 years (n=577), while in men it was 75-79 years (n=885). It is noteworthy that in 1990, the number of deaths in all age groups was higher in women than in men, and that the number of deaths in most age groups began to exceed that of women of the same age in 2021. Similarly, with regard to DALYs, **Figure 4D** illustrates that in 1990, the age group with the highest number of DALYs in women was 60-64 years (n=8, 601), while in men it was 55-59 years (n=6, 563). Similarly, **Figure 4H** illustrates that in 2021, the age group with the highest number of deaths in women was 65-69 years (n=14, 367), while in men it was 55-59 years (n=19, 360). It is noteworthy that in 1990, DALYs in all age groups were higher in women than in men, and in 2021, the DALYs in most age groups of men began to be higher in men than in women.

**Figure 5:**
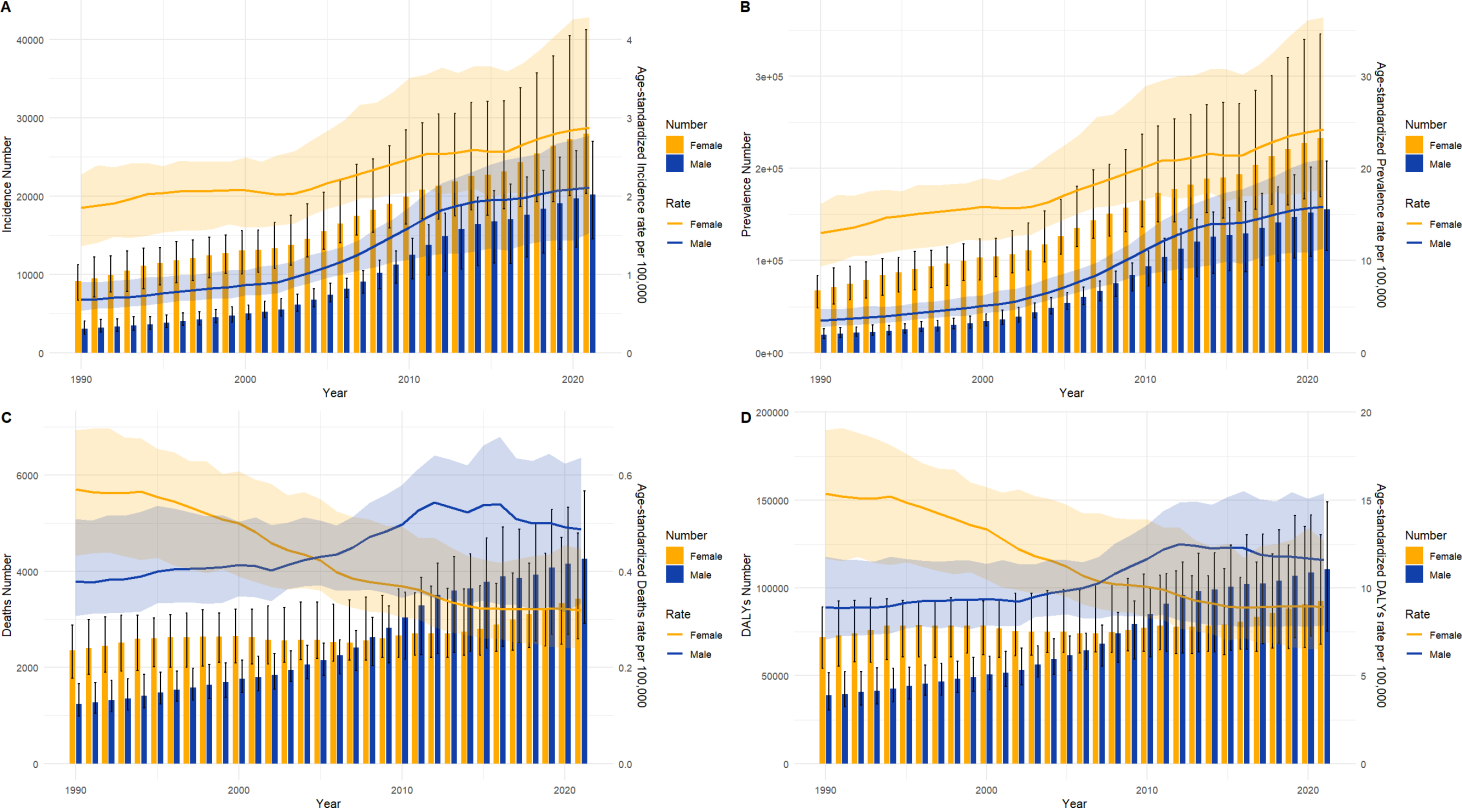
Full-age numbers and age-standardized rates of incidence, prevalence, deaths and DALYs among TC males and females in China from 1990 to 2021. **(A)** Incidence number and ASIR; **(B)** Prevalence number and ASPR; **(C)** Death number and ASDR; **(D)** DALYs and ASDALYs rates. lines represent age-standardized rates; Bar charts represent Counts.

**Figure 5** presents a comparison of the disease burden and age-standardized rates of TC in men and women across all age groups in China from 1990 to 2021. **Figure 5A** illustrates that the ASIR and incidence of TC in women and men increased over time, with smaller gender differences. Similarly, **Figure 5B** depicts that the number of women and men with ASPR and the prevalence of TC also increased over the years, and that ASPR was consistently higher in women than in men. **Figure 5C** illustrates the trend of ASDR and death in TC in women and men over the years. It is noteworthy that the ASDR of TC in women decreased year by year, contrary to men. Furthermore, the ASPR of TC in men began to exceed that of women after 2004. **Figure 5D** illustrates the ASDALYs rate and DALYs in women and men for TC over time. As with the ASDR, after 2007, the ASPR for TC in men began to exceed that in women. Furthermore, after 2015, the ASDALYs rate in both women and men began to stabilize.

### Age-period-cohort effects on prevalence and death of TC in China

**Figure 6:**
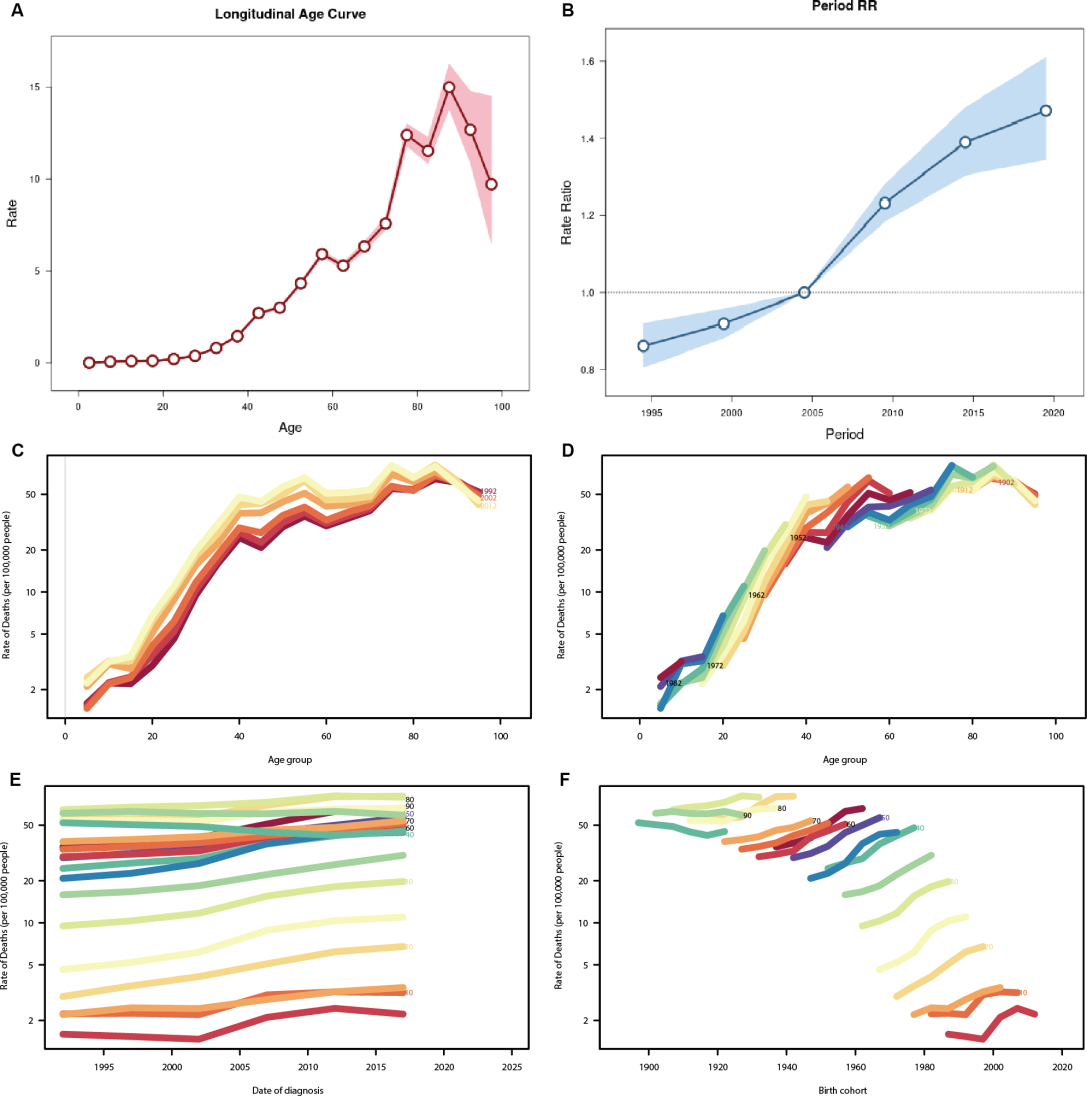
Age-period-cohort analysis for prevalence and death rate of TC in China. **(A)** Age effect for prevalence rate, showing the relation between prevalence and age **(B)** Period effect for prevalence rate, which shows the relationship between prevalence and cycle, defining the relative prevalence rate during the time period from 2002 to 2007 as 1. **(C)** The age-specific death rate according to time periods; each line connects the age-specific death rate for a 5-year period. **(D)** The age-specific death rate according to birth cohorts; each line connects the age-specific mortality for a 5-year cohort. **(E)** The period-specific death rate according to age group; each line connects the birth cohort-specific deathrate for a 5-year age group. **(F)** The birth cohort-specific death rate according to age groups; each line connects the birth cohort-specific deathrate for a 5-year age group.

**Figure 6** illustrates the age-period-cohort effect of TC prevalence and death in China. Based on the results of the Ward test, the fitted age-period-cohort model is deemed to be significant (P<0.05). **Figure 6A** illustrates the relationship between prevalence and age. It is evident that prevalence generally increases with age; however, it is noteworthy that there is a sharp decline in prevalence after the age of 85-89 years (Rate=14.991, 95%CI=13.827-16). **Figure 6B** illustrates the relationship between prevalence and period, with the prevalence from 2002 to 2006 set at 1 and the relative prevalence at other times displayed. It can be seen that the relative prevalence rate increased with each passing year, from 1992 to 1996 (RR=0.861, 95%CI=0.806-0.091) to 2017-2021 (RR=1.471, 95%CI=1.345-1.609). **Figure 6C** illustrates the trends in death cohorts by age distribution during specific periods, including 1992, 2002, and 2012. It can be observed that the death rate increases most rapidly between the ages of 20 and 40. **Figure 6D** provides supplementary information regarding cohort trends in death by age group. **Figure 6E** illustrates the temporal trend of TC death based on diagnostic cohorts from 1992 to 2021. The data indicate that death rates increase slightly with age at diagnosis in the majority of age groups. Adolescents exhibit lower death rates, while older and middle-aged individuals display higher death rates. **Figure 6F** illustrates the temporal trend of death based on birth cohorts, demonstrating a gradual increase in TC death rates across age-specific cohorts as the age at birth progresses. It is noteworthy that, although the death rate of young adults is considerably lower than that of middle-aged and older individuals, their death rates exhibit a more pronounced increase with age at birth than those of middle-aged and older individuals.

### Decomposition analysis of incidence and death of TC in China and forecast in the next decade

**Figure 7:**
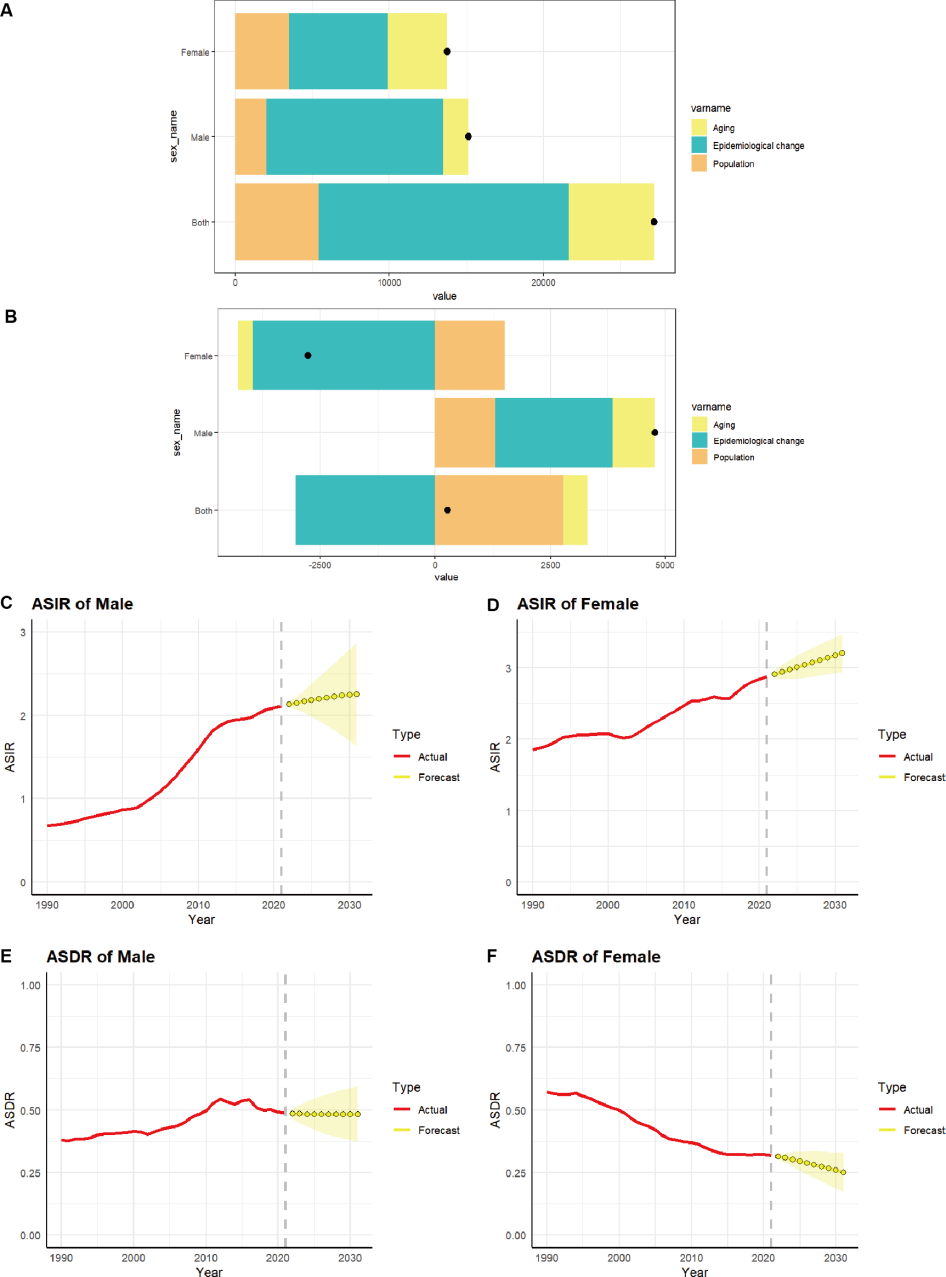
The relative contributions of aging, epidemiological changes, and population to the number of incidence **(A)** and deaths **(B)** of TC from 1990 to 2021 in China. The black dot represents the overall value of change contributed by all 3 components from 1990 to 2021. **(C–F)** Predicted trends of thyroid cancer ASIR and ASDR by sex in China over the next 10 years (2021–2031). Red lines represent the true trend of thyroid cancer ASIR and ASDR during 1990–2021; Yellow dot lines and shaded regions represent the predicted trend and its 95% CI.

Decomposition analysis was employed to ascertain the proportional impact of aging, epidemiological shifts, and population growth on the incidence (**Figure 7A**) and deaths (**Figure 7B**) of TC in China. **Table 2** illustrates that the rise in TC number in China is predominantly attributable to epidemiological changes, accounting for 59.66% of the total. Of this, 46.61% are females and 76.1% are males. With regard to death, the decline in the number of female deaths was predominantly attributable to epidemiological changes, accounting for 143.52%. Conversely, the slight increase in the overall number of deaths in the population was primarily attributed to the impact of aging and population growth exceeding the influence of epidemiological changes.

**Table 2:** TC Change due to aging, epidemiological changes, and population by sex (“%” means contribution to the total changes).

Burden index	Sex	Overall difference	Aging	Epidemiological changes	Population
Incidence	Female	13761.13	3876.056 (28.17%)	6414.013 (46.61%)	3471.058 (25.22%)
	Male	15129.78	1616.321 (10.68%)	11513.439 (76.1%)	2000.016 (13.22%)
	Both	27185.35	5571.274 (20.49%)	16219.758 (59.66%)	5394.32 (19.84%)
Deaths	Female	-2764.46	-307.605 (194.25%)	-3967.263 (-1112.75%)	1510.411 (1018.5%)
	Male	4775.57	913.052 (19.12%)	2562.606 (53.66%)	1299.909 (27.22%)
	Both	272.95	530.222 (11.13%)	-3037.263 (143.51%)	2779.987 (-54.64%)

The ARIMA model was employed to provide a quantitative description of the trends in the ASIR and ASDR of TC in Chinese women and men over the subsequent decade. Following filtering by the auto.arima() function, the optimal model for the incidence of TC males was identified as “1,1,1” (σ^2^ = 2.353e-04, AIC = -164.39, BIC = -160.08). The optimal model for the incidence of TC women was “0,1,1” (σ^2^ = 7.18e-04, AIC= -131.96, BIC= -127.66). The optimal model for the deaths of TC males was “1,1,0” (σ^2^ = 1.135e-04, AIC= -190.4, BIC= -187.53). The optimal model for the deaths of TC females was “1,1,0” (σ^2^ = 2.736e-05, AIC= -233.26, BIC= -228.96). It is predicted that the ASIR for men (**Figure 7C**) and women (**Figure 7D**) will gradually increase over the next 10 years, with an ASIR of 2.25 per 100,000 population for men and 3.20 per 100,000 population for women by 2031. The ASDR for men (**Figure 7E**) is expected to stabilize over the next decade, remaining above 0.48 per 100,000 population. In contrast, the ASDR for women (**Figure 7F**) is projected to decline gradually over the same period, with an ASDR of 0.25 per 100,000 population for women anticipated by 2031.

## Discussion

This study presents a comprehensive evaluation of the incidence, prevalence, death, and DALYs associated with TC in China and worldwide over the past 32 years, based on data from the GBD 2021 database. A comparison of the burden of TC by age and sex in China was also conducted. The findings revealed that from 1990 to 2021, the ASIR and ASPR of TC in China and worldwide increased, while the ASDR and ASDALYs rate both decreased. The disease burden of TC is associated with patient age, with a high incidence in middle-aged individuals and a high death rate in older individuals. This is supported by the findings of a retrospective analysis, which identified age as an independent risk factor for TC (20). Hu et al. (21) predicted through the GBD 2019 database that the global burden of TC is increasing, particularly among females and in regions with medium-to-low SDI. The findings revealed that women are more susceptible to developing TC than men. This is generally consistent with the findings of our study. However, the death rate in men surpassed that in women at the beginning of the 21st century. This is evidenced by the significant rise in the number of cases and deaths in older men. This disparity may be attributed to the poor prognosis of TC in older men, the advanced detection of the disease, and the higher degree of malignancy and aggressiveness of the tumor (22). TC is the sole non-reproductive cancer that predominantly affects female patients. In light of the established link between autoimmune thyroid disease and TC risk (23), the elevated prevalence of TC in women may be attributable to the underlying causes of the female predominance in autoimmune thyroid disease (24). Furthermore, estrogen has been demonstrated to facilitate the proliferation and invasion of TC (25–27).

The diagnosis of thyroid nodules has been the subject of continuous development, and the resolution of ultrasound for the diagnosis of nodules has also improved. The widespread availability of the Ultrasound Thyroid Imaging Reporting and Data System (TIRADS) and ultrasound fine needle aspiration biopsy (FNB) technology has resulted in an increased incidence and prevalence of TC in China and worldwide (28). This may be attributed to overdiagnosis. An analysis conducted by researchers from the International Agency for Research on Cancer (IARC) revealed that the estimated proportion of TC cases in women attributed to overdiagnosis is 87% in China, which is higher than in men (29). This finding may also elucidate the gender disparity observed in TC incidence. Furthermore, it is crucial to consider the rising prevalence of risk factors such as genetics, obesity, radiation exposure, and even betel nut consumption (2). However, the enhancement of early diagnosis indirectly enhances the survival prognosis of TC patients. a multicenter study had shown that early diagnosis can effectively improve the disease-free survival of patients with DTC (30). Furthermore, the development of immunotherapy and targeted therapy for ATC has also been effective in alleviating the disease burden of TC (31, 32). The strategy of combining anti-PD-L1 immunotherapy with mutation-driven targeted therapy has been proven to effectively extend the median overall survival in patients with ATC (33). The reduction in deaths and DALYs in China and worldwide can be attributed to advances in these prevention and treatment measures.

Chen et al. (34) utilized the APC model to analyze the GBD 2019 database, revealing that globally, the impact of age was most significant in individuals aged 95+, while it was minimal in those aged 0-14. The period effect demonstrated a relatively low risk of prevalence during the period from 1990 to 2004, followed by a higher relative risk of prevalence from 2005 to 2019. Furthermore, the cohort effect indicated a lower relative risk of developing the disease before 1950 and a higher risk thereafter. Our findings indicate that the age effect and period effect exert a significant influence on the prevalence of TC in China, particularly among middle-aged and elderly individuals, whose prevalence is notably high. However, the prevalence of elderly patients is on the decline. The relative prevalence rate has continued to increase throughout the course of the cycle. In the cohort effect, we also observed a lower death risk among earlier birth cohorts, and it may be attributed to the accelerated process of population aging.

It is notable that Chinese aging population has resulted in an increase in TC deaths among men, while a reduction in TC deaths has been observed among women. This may be related to the decline in estrogen levels in women following perimenopause. The monitoring of disease epidemiology and the prediction of trends represent a crucial aspect of disease prevention and control. The ARIMA model indicates that the ASIR for both male and female patients with TC will increase gradually over the next decade, which is consistent with the findings of a previous study (34). Conversely, the ASDR for male patients is expected to stabilize, while that for female patients will continue to decline. It can be observed that the incidence of TC in China is increasing, the age at which patients are most likely to be diagnosed has shifted towards younger age groups, and the death rate has demonstrated a tendency to stabilize or even decline. There are notable variations in the incidence of TC across different countries and regions globally, with the incidence in women varying by up to 15 times (35). The National Cancer Center of China has reported a significant increase in the incidence rate of TC among Chinese women in recent years (36), which is consisted with the result of our study. However, the death rates of TC patients remain relatively low. A review of the literature reveals that for individuals diagnosed with papillary TC and exhibiting a lesion diameter of less than 1 cm, active monitoring and surgical intervention only when the tumor progresses (either in terms of increased size or the emergence of suspicious lymph nodes) can achieve outcomes comparable to those of immediate surgical treatment (37). Since 2015, there has been a significant decline in the incidence rate of TC in South Korea during the same period, accompanied by an increase in cancer-specific mortality among patients (38). From 2000 to 2017, the average annual increase in thyroid cancer-specific mortality in the United States was 1.7% (39). These results from developed countries differ from the trends in ASDR we have seen in China and worldwide. Additionally, we found the ASDR in men has been on the rise over the past two decades, declining in recent years, and is expected to stabilize over the next decade. This may be attributed to the advancement of immunotherapy and targeted therapy in ATC.

This study still has some limitations. Firstly, although the GBD adjusted data sources in 2021 to account for the heterogeneity of different studies and standardize the data, these revisions have increased the uncertainty of the data analyzed. Secondly, the majority of the data sources are cross-sectional studies, which may result in an overestimation of the prevalence of TC. The GBD database only includes crude death rates for TC, lacking data on cancer-specific death rate. While this is more conducive to assessing the overall impact of TC on patient survival, it slightly lacks precision. Meanwhile, due to the inability to obtain pathological biopsy data, there is a lack of trend analysis on the burden of various pathological subtypes of TC in China. Furthermore, the geographic description of the burden of TC in China was lacking due to the unavailability of both provincial data and rural-urban data. The age-period-cohort analysis reveals results at the population level, which may be susceptible to ecological fallacies. Furthermore, the projected outcomes for the period between 2022 and 2031 are contingent upon the outputs of the predictive model. Consequently, these projections may undergo revision in light of advancements in data quality and methodological enhancements. Finally, the level of disease burden in different countries or regions may vary depending on a number of factors, including socio-economic development, geographical environmental factors, the level of medical resources available, genetics, ethnicity, and so forth. It should be noted that the global data may not be directly applicable to specific reference levels of disease burden in a particular country or region. Consequently, an in-depth analysis is required based on the specific data of each country or region.

## Conclusion

The incidence and prevalence of “TC” in China has increased markedly in recent years, with a discernible trend towards the global average. The strategy for the early screening of TC in China should be more targeted and forward-looking, with the aim of avoiding unnecessary screening, diagnosis, and treatment. This will help to reduce the burden on the medical and health system, as well as on patients. Furthermore, it is imperative that researchers devote sufficient attention to the fundamental mechanisms, early diagnosis and clinical treatment of TC with a poor prognosis in order to effectively reduce the burden of TC death. In light of the country’s rapidly aging population and the concomitant improvement in socio-economic status, TC remains a significant public health challenge in China, necessitating a careful balance of the cost-benefit ratio.

## Data Availability

Publicly available datasets were analyzed in this study. This data can be found here: The Global Burden of Disease study 2021 is an open-access resource; data are available at http://ghdx.healthdata.org/gbd-results-tool.

## References

[1] Pstrąg N Ziemnicka K Bluyssen H Wesoły J. Thyroid cancers of follicular origin in a genomic light: in-depth overview of common and unique molecular marker candidates. Mol Cancer. (2018) 17:116. doi: 10.1186/s12943-018-0866-1 30089490 PMC6081953

[2] Kim J Gosnell JE Roman SA. Geographic influences in the global rise of thyroid cancer. Nat Rev Endocrinol. (2020) 16:17–29. doi: 10.1038/s41574-019-0263-x 31616074

[3] Lim H Devesa SS Sosa JA Check D Kitahara CM. Trends in thyroid cancer incidence and mortality in the United States, 1974-2013. JAMA. (2017) 317:1338–48. doi: 10.1001/jama.2017.2719 PMC8216772 28362912

[4] Miranda-Filho A Lortet-Tieulent J Bray F Cao B Franceschi S Vaccarella S. Thyroid cancer incidence trends by histology in 25 countries: a population-based study. Lancet Diabetes Endocrinol. (2021) 9:225–34. doi: 10.1016/S2213-8587(21)00027-9 33662333

[5] Siegel RL Miller KD Wagle NS Jemal A. Cancer statistics, 2023. CA Cancer J Clin. (2023) 73:17–48. doi: 10.3322/caac.21763 36633525

[6] Kent WD Hall SF Isotalo PA Houlden RL George RL Groome PA. Increased incidence of differentiated thyroid carcinoma and detection of subclinical disease. CMAJ. (2007) 177:1357–61. doi: 10.1503/cmaj.061730 PMC2072986 18025426

[7] Cabanillas ME McFadden DG Durante C. Thyroid cancer. Lancet. (2016) 388:2783–95. doi: 10.1016/S0140-6736(16)30172-6 27240885

[8] Veiga LH Neta G Aschebrook-Kilfoy B Ron E Devesa SS. Thyroid cancer incidence patterns in Sao Paulo, Brazil, and the U.S. SEER program, 1997-2008. Thyroid. (2013) 23:748–57. doi: 10.1089/thy.2012.0532 PMC3675840 23410185

[9] Wang Y Wang W. Increasing incidence of thyroid cancer in Shanghai, China, 1983-2007. Asia Pac J Public Health. (2015) 27:NP223–9. doi: 10.1177/1010539512436874 22345304

[10] Ahn HS Kim HJ Kim KH Lee YS Han SJ Kim Y. Thyroid cancer screening in South Korea increases detection of papillary cancers with no impact on other subtypes or thyroid cancer mortality. Thyroid. (2016) 26:1535–40. doi: 10.1089/thy.2016.0075 27627550

[11] Uhry Z Colonna M Remontet L Grosclaude P Carré N Couris CM. Estimating infra-national and national thyroid cancer incidence in France from cancer registries data and national hospital discharge database. Eur J Epidemiol. (2007) 22:607–14. doi: 10.1007/s10654-007-9158-6 17636414

[12] Deng Y Li H Wang M Li N Tian T Wu Y. Global burden of thyroid cancer from 1990 to 2017. JAMA Netw Open. (2020) 3:e208759. doi: 10.1001/jamanetworkopen.2020.8759 32589231 PMC7320301

[13] Zhai M Zhang D Long J Gong Y Ye F Liu S. The global burden of thyroid cancer and its attributable risk factor in 195 countries and territories: A systematic analysis for the Global Burden of Disease Study. Cancer Med. (2021) 10:4542–54. doi: 10.1002/cam4.3970 PMC8267141 34002931

[14] Rahbari R Zhang L Kebebew E. Thyroid cancer gender disparity. Future Oncol. (2010) 6:1771–9. doi: 10.2217/fon.10.127 PMC3077966 21142662

[15] Shobab L Burman KD Wartofsky L. Sex differences in differentiated thyroid cancer. Thyroid. (2022) 32:224–35. doi: 10.1089/thy.2021.0361 34969307

[16] Wiltshire JJ Drake TM Uttley L Balasubramanian SP. Systematic review of trends in the incidence rates of thyroid cancer. Thyroid. (2016) 26:1541–52. doi: 10.1089/thy.2016.0100 27571228

[17] Di Cristofano A. The year in basic thyroid cancer research. Thyroid. (2022) 32:3–8. doi: 10.1089/thy.2021.0561 34806425 PMC8792493

[18] Zhou T Wang X Zhang J Zhou E Xu C Shen Y. Global burden of thyroid cancer from 1990 to 2021: a systematic analysis from the Global Burden of Disease Study 2021. J Hematol Oncol. (2024) 17:74. doi: 10.1186/s13045-024-01593-y 39192360 PMC11348565

[19] Cheng F Xiao J Shao C Huang F Wang L Ju Y. Burden of thyroid cancer from 1990 to 2019 and projections of incidence and mortality until 2039 in China: findings from global burden of disease study. Front Endocrinol (Lausanne). (2021) 12:738213. doi: 10.3389/fendo.2021.738213 34690931 PMC8527095

[20] Wang Y Wang J Chen Z Ma M Lin C He Q. Analysis of the correlation between high iodized salt intake and the risk of thyroid nodules: a large retrospective study. BMC Cancer. (2021) 21:1000. doi: 10.1186/s12885-021-08700-z 34493230 PMC8425165

[21] Hu S Wu X Jiang H. Trends and projections of the global burden of thyroid cancer from 1990 to 2030. J Glob Health. (2024) 14:4084. doi: 10.7189/jogh.14.04084 PMC11109522 38751316

[22] Girardi FM. Thyroid carcinoma pattern presentation according to age. Int Arch Otorhinolaryngol. (2017) 21:38–41. doi: 10.1055/s-0036-1585095 28050206 PMC5205525

[23] Fiore E Rago T Latrofa F Provenzale MA Piaggi P Delitala A. Hashimoto’s thyroiditis is associated with papillary thyroid carcinoma: role of TSH and of treatment with L-thyroxine. Endocr Relat Cancer. (2011) 18:429–37. doi: 10.1530/ERC-11-0028 21565972

[24] Simmonds MJ Kavvoura FK Brand OJ Newby PR Jackson LE Hargreaves CE. Skewed X chromosome inactivation and female preponderance in autoimmune thyroid disease: an association study and meta-analysis. J Clin Endocrinol Metab. (2014) 99:E127–31. doi: 10.1210/jc.2013-2667 24187400

[25] Wang Q Huang H Zhao N Ni X Udelsman R Zhang Y. Phytoestrogens and thyroid cancer risk: A population-based case-control study in connecticut. Cancer Epidemiol Biomarkers Prev. (2020) 29:500–8. doi: 10.1158/1055-9965.EPI-19-0456 PMC7007342 31826911

[26] Zhang L Zhou M Gao X Xie Y Xiao J Liu T. Estrogen-related genes for thyroid cancer prognosis, immune infiltration, staging, and drug sensitivity. BMC Cancer. (2023) 23:1048. doi: 10.1186/s12885-023-11556-0 37907864 PMC10619281

[27] Xu FZ Zheng LL Chen KH Wang R Yi DD Jiang CY. Serum sex hormones correlate with pathological features of papillary thyroid cancer. Endocrine. (2024) 84:148–54. doi: 10.1007/s12020-023-03554-w PMC10987349 37815746

[28] Hsiao V Massoud E Jensen C Zhang Y Hanlon BM Hitchcock M. Diagnostic accuracy of fine-needle biopsy in the detection of thyroid Malignancy: A systematic review and meta-analysis. JAMA Surg. (2022) 157:1105–13. doi: 10.1001/jamasurg.2022.4989 PMC9558056 36223097

[29] Li M Dal Maso L Vaccarella S. Global trends in thyroid cancer incidence and the impact of overdiagnosis. Lancet Diabetes Endocrinol. (2020) 8:468–70. doi: 10.1016/S2213-8587(20)30115-7 32445733

[30] Díez JJ Anda E Alcazar V Isidro ML Familiar C Paja M. Differentiated thyroid carcinoma in the elderly: influence of age on disease-free and overall survival. Endocrine. (2022) 77:121–33. doi: 10.1007/s12020-022-03059-y 35585463

[31] Gainor JF Curigliano G Kim DW Lee DH Besse B Baik CS. Pralsetinib for RET fusion-positive non-small-cell lung cancer (ARROW): a multi-cohort, open-label, phase 1/2 study. Lancet Oncol. (2021) 22:959–69. doi: 10.1016/S1470-2045(21)00247-3 34118197

[32] Maniakas A Zafereo M Cabanillas ME. Anaplastic thyroid cancer: new horizons and challenges. Endocrinol Metab Clin North Am. (2022) 51:391–401. doi: 10.1016/j.ecl.2021.11.020 35662448

[33] Cabanillas ME Dadu R Ferrarotto R Gule-Monroe M Liu S Fellman B. Anti-programmed death ligand 1 plus targeted therapy in anaplastic thyroid carcinoma: A nonrandomized clinical trial. JAMA Oncol. (2024), e244729. doi: 10.1001/jamaoncol.2024.4729 39446377 PMC11581602

[34] Chen J Wang C Shao B. Global, regional, and national thyroid cancer age-period-cohort modeling and Bayesian predictive modeling studies: A systematic analysis of the global burden of disease study 2019. Heliyon. (2023) 9:e22490. doi: 10.1016/j.heliyon.2023.e22490 38045179 PMC10689957

[35] Pizzato M Li M Vignat J Laversanne M Singh D La Vecchia C. The epidemiological landscape of thyroid cancer worldwide: GLOBOCAN estimates for incidence and mortality rates in 2020. Lancet Diabetes Endocrinol. (2022) 10:264–72. doi: 10.1016/S2213-8587(22)00035-3 35271818

[36] Lamartina L Leboulleux S Borget I Schlumberger M. Global thyroid estimates in 2020. Lancet Diabetes Endocrinol. (2022) 10:235–6. doi: 10.1016/S2213-8587(22)00048-1 35271820

[37] Zheng R Zhang S Zeng H Wang S Sun K Chen R. Cancer incidence and mortality in China, 2016. J Natl Cancer Cent. (2022) 2:1–9. doi: 10.1016/j.jncc.2022.02.002 39035212 PMC11256658

[38] Kim KJ Choi J Park SK Park YJ Kim SG. Thyroid cancer-specific mortality during 2005-2018 in Korea, aftermath of the overdiagnosis issue: a nationwide population-based cohort study. Int J Surg. (2024) 110:5489–95. doi: 10.1097/JS9.0000000000001767 PMC11392158 38874484

[39] Yan KL Li S Tseng CH Kim J Nguyen DT Dawood NB. Rising incidence and incidence-based mortality of thyroid cancer in California, 2000-2017. J Clin Endocrinol Metab. (2020) 105:dgaa121. doi: 10.1210/clinem/dgaa121 32166320

